# Global vs. Network-Specific Regulations as the Source of Intrinsic Coactivations in Resting-State Networks

**DOI:** 10.3389/fnsys.2019.00065

**Published:** 2019-10-29

**Authors:** Shiori Amemiya, Hidemasa Takao, Osamu Abe

**Affiliations:** Department of Radiology, Graduate School of Medicine, The University of Tokyo, Tokyo, Japan

**Keywords:** fMRI, resting-state network, spatiotemporal dynamics, spontaneous neural activity, neuronal pathway tracing

## Abstract

Spontaneous neural activities are endowed with specific patterning characterized by synchronizations within functionally relevant distant regions that are termed as resting-state networks (RSNs). Although the mechanisms that organize the large-scale neural systems are still largely unknown, recent studies have proposed a hypothesis that network-specific coactivations indeed emerge as the result of globally propagating neural activities with specific paths of transmission. However, the extent to which such a centralized global regulation, rather than network-specific control, contributes to the RSN synchronization remains unknown. In the present study, we investigated the contribution from each mechanism by directly identifying the global as well as local component of resting-state functional MRI (fMRI) data provided by human connectome project, using temporal independent component analysis (ICA). Based on the spatial distribution pattern, each ICA component was classified as global or local. Time lag mapping of each IC revealed several paths of global or semi-global propagations that are partially overlapping yet spatially distinct to each other. Consistent with previous studies, the time lag of global oscillation, although being less spatially homogenous than what was assumed to be, contributed to the RSN synchronization. However, an equivalent contribution was also shown on the part of the more locally confined activities that are independent to each other. While allowing the view that network-specific coactivation occurs as part of the sequences of global neural activities, these results further confirm an equally important role of the network-specific regulation for its coactivation, regardless of whether vascular artifacts contaminate the global component in fMRI measures.

## Introduction

Once considered to be a noisy, stochastic process, spontaneous activity of the cortical neuron is now understood to be by no means random but is endowed with specific patterning that reflects the functional architecture of the underlying network at the level of micro- or meso-circuits (Tsodyks et al., 1999; Kenet et al., 2003). Over the last two decades, it has become apparent that this is analogously true at the level of large-scale networks that are defined using resting-state functional magnetic resonance imaging (rs-fMRI) (Biswal et al., 1995; Fox and Raichle, 2007). The spatial patterns identified as areas with synchronous oscillation of the blood oxygenation-level dependent (BOLD) signal are termed as resting-state networks (RSNs) (Fox et al., 2005). These networks are closely related to anatomical connectivity among the neural subsystems that have been revealed by a wide variety of visual, sensorimotor, and cognitive task paradigms (Vincent et al., 2007; Zhang et al., 2010). However, the neurophysiology of the phenomenon, or the mechanism that controls and coordinates the intrinsic synchronization across distributed neural systems, largely remains to be established.

While conventional rs-fMRI analyses based on seed-based correlation or independent component analysis (ICA) implicitly assume that the spatial distribution of the synchronous neural activity is temporally constant, animal studies have revealed that spontaneous neural activity is spatiotemporally structured, and propagating waves of activity have been recorded in a variety of species (for review, see Muller and Destexhe, 2012). Neuronal membrane potential in the cortex undergoes a spontaneous transition between up and down states in the absence of sensory inputs (Steriade et al., 1993; Lampl et al., 1999; Petersen et al., 2003; Shu et al., 2003). Population activity of the neurons during the up state manifests as propagating waves not only within a part of the cortex (Petersen et al., 2003; Ferezou et al., 2007; Xu et al., 2007; Civillico and Contreras, 2012), but also throughout the entire brain (Stroh et al., 2013). The spatiotemporal dynamics of the low-frequency oscillation have also been identified by examining the repetitive spatiotemporal patterns (Majeed et al., 2009, 2011; Takeda et al., 2016; Belloy et al., 2018; Abbas et al., 2019) or by analyzing the time lag structures of the rs-fMRI data (Mitra et al., 2014, 2015a; Amemiya et al., 2016; Matsui et al., 2016). An intriguing hypothesis proposed by one of those studies is that the RSN synchronization indeed emerges as the result of several independent global propagations of spontaneous neural activity (Mitra et al., 2015a). Using synthetic time series embedded with the measured time lag structures of the rs-fMRI data, Mitra et al. (2015a) showed that the functional connectivity (FC) matrix representing the RSN synchronization could be reconstructed to a fair approximation. In support of this idea, a recent animal study also showed that a global wave of spontaneous neuronal activity propagating across the networks contributes to within-network coactivations of the neurons that correspond to RSN synchronization (Matsui et al., 2016). Based on these findings it follows that seemingly independent RSN activity can be viewed as being controlled by a single centralized mechanism, through global wave(s) of activity that regulate and constrain the relative relationships of the network activity by determining the order and timing of the activation of each network. However, it remains unclear if this is the sole mechanism that gives rise to the RSN synchronization, as suggested by those studies (Mitra et al., 2015b; Matsui et al., 2016). An alternative, thought non-exclusive, origin of the synchronization would be network-specific coactivations among the neural populations confined within each network (Mohajerani et al., 2013; Ponce-Alvarez et al., 2015). In the neural system, it is generally supposed that diverse physiological mechanisms coexist for rhythm generation and population synchronization for which different levels of integration interact closely with each other (Ivanchenko et al., 2008; Harris-Warrick, 2010; Wang, 2010). For example, in the respiratory central pattern generator of the mammals, rhythm generation is dependent on the endogenously oscillatory neurons that serve as pacemaker, as well as the pattern of synaptic connections within the network that forms a network pacemaker (hybrid pacemaker-network mechanism) (Calabrese, 1998; Rybak et al., 2004, 2007; Sohal et al., 2006; Johnson et al., 2007).

It seems possible, therefore, that multiple mechanisms – namely, global propagation and local synchronization – contribute to the emergence of the coherent RSN activity that characterizes the functional architecture of the brain. In order to address this question, it is imperative to evaluate not only the paths but also the whole picture of the traveling waves. We thus started by identifying the signal time course of the global waves by applying the temporal ICA to rs-fMRI data. In fMRI, virtually all applications of ICA use spatial rather than temporal ICA. Although spatial ICA is suitable for the separation of the spatially distinct activations from each other, temporal ICA would be more appropriate if the aim is to find functionally independent and spatially overlapping activities (Smith et al., 2012), such as what we assume to be multiple global waves.

In contrast to previous studies focusing on estimating the paths of traveling waves by analyzing the signal time lag (Mitra et al., 2014, 2015a; Amemiya et al., 2016; Matsui et al., 2016), direct detection of the traveling waves enables us to infer the likelihood that each mechanism contributes to the emergence of network synchronization, as well as to map the magnitude of each type of activity in each region. Moreover, identification of individual traveling waves allows accurate estimation of the time lag structures, in contrast to previous studies employing a decomposition approach, regardless of the validity of the assumptions that the time lags of multiple waves can be linearly superposed, or that the paths of traveling waves are spatially independent. By comparing the correlation matrix of both the global and the local component to that of the FC matrix, contribution of each type of activity to the RSN synchronization that characterizes the resting state FC was evaluated.

## Materials and Methods

### Overview

**FIGURE 1:**
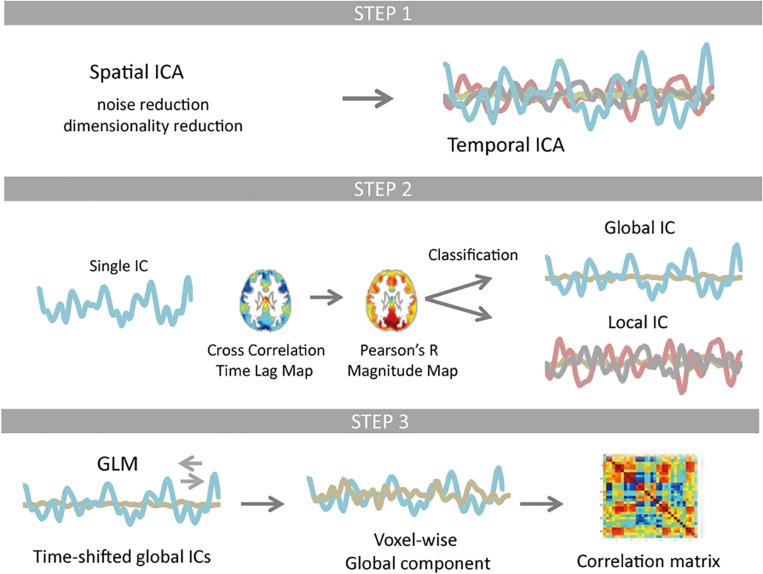
Schematic of the resting-state fMRI data processing. Temporal independent component analysis (ICA) was applied to pre-processed test and re-test dataset, whose dimensionality were reduced to 61 × 120,000 and 62 × 120,000 using spatial ICA, respectively. Each temporal ICA gave 28 and 30 reproducible components (Step 1). For each component (IC time series), a time lag map was obtained by computing the time lag of each voxel relative to the IC time series using cross-correlation, which was further interpolated with parabolic polynomials. Pearson’s correlation coefficients were computed between each voxel’s time series and the IC time series that was shifted as much as the measured time lag. Classification of ICs was based on spatial distribution pattern of each component. Any component that is more similar to the whole-brain signal than any RSN template in distribution pattern was classified as global (Step 2). Using the time-shifted global ICs as regressors, linear regression analysis was performed for each voxel. All global ICs detected for any single temporal ICA were then integrated. The rest of the signal change was classified as local contribution. The correlation matrix of the global as well as local component was computed and compared with the correlation matrix of the original signal FC matrix (Step 3).

A summary of the analysis is presented as a schematic in Figure 1 to provide an overview of the study. We used data from the WU-Minn Human Connectome Project (HCP) young healthy adults (ages 22–35) S1200 release that provides paired dataset of the same group of subjects (day 1 and day 2). All preprocessing and data analyses were performed for each dataset, respectively, in the same way.

### HCP Data

Data of 50 subjects who underwent 3 T resting-state fMRI sessions without quality control issues, and whose mean framewise displacement was less than 0.2 mm were included for the analysis. The number of subject was determined by the maximum size of the data that could be processed by a core program for ICA, Multivariate Exploratory Linear Decomposition into Independent Components (MELODIC) (Beckmann and Smith, 2004). HCP imaging and pre-processing protocol have been previously described in detail (Glasser et al., 2013; Smith et al., 2013; Van Essen et al., 2013; Griffanti et al., 2014; Salimi-Khorshidi et al., 2014). In brief, resting-state fMRI data were acquired using a single customized Siemens 3 T scanner housed at Washington University in St. Louis, using a standard 32-channel receive head coil, with 2.0 mm isotropic spatial resolution, 0.72 s repetition time (TR), and 1200 frames, i.e., 14.4 min per run. For each subject, and for each session, two runs with reversed phase encoding directions, RL or LR, with the order counterbalanced across each of two sessions, were acquired (WU-Minn HCP 1200 Subjects Data Release Reference Manual), and the geometric distortions were corrected using spin echo field map EPI scans (Glasser et al., 2013). The data were then subjected to spatial ICA using MELODIC with automatic dimensionality estimation. Using FMRIB’s ICA-based X-noisifier (FIX) (Griffanti et al., 2014; Salimi-Khorshidi et al., 2014) that is a machine learning classifier trained on HCP data, spatially specific noise components were identified and removed for each run. Then 24 movement regressors were further regressed out of the data (Smith et al., 2013; Griffanti et al., 2014; Salimi-Khorshidi et al., 2014).

### Data Analysis

#### Further Pre-processing

Further pre-processing and analysis of the data were performed using tools from SPM12 software^1^, AFNI libraries^2^ and in-house scripts written and implemented in Matlab 9.3 (MathWorks, Natick, MA, United States). Linear trends were removed from the HCP data that had been processed with subject-level ICA noise reduction (sICA + FIX), and the data were band-pass filtered at 0.01–0.1 Hz. The pre-processed data were temporally concatenated across runs to create a single 4D dataset of 120,000 timepoints for test and re-test dataset, respectively.

#### Temporal-ICA

For temporal ICA decomposition of the data, we employed a strategy adapted from Smith et al. (2012) to perform group-wise spatial ICA in advance of the final temporal ICA. The spatial ICA allows further identification of artifact components at the group level, as well as to achieve a high-dimensional functional parcelation of the group data, which reduces the dimensionality of the data to feed into temporal ICA. The overall ICA analysis is described as follows:

$$X_{\left(V\times T\right)}=\,S_{S\,\left(V\times K\right)}\times A_{t\,\left(K\times L\right)}\times S_{t\,\left(L\times T\right)}+E$$

where *X* is the data matrix of size *V*oxels × *T*ime points, *S*_*s*_ are the spatial maps estimated by spatial ICA, *K* is the number of spatial ICA components that were subsequently fed into temporal ICA, after removing noise components. *L* is the number of temporal ICA components. *S*_*t*_ is the decomposed time series (ICA sources), and *A*_*t*_ is the central mixing matrix of temporal ICA. *E* combines noise and artifact aspects of the data (Smith et al., 2012).

Group-wise spatial ICA was performed using MELODIC with automatic dimensionality estimation. Of the 61 and 62 ICs generated from dataset 1 and 2, three ICs were classified as artifacts on the basis of their spatial features, respectively. Specifically, activation patterns clearly outlining the intensity edges of the gray matter were classified as noise (Smith et al., 2012; Salimi-Khorshidi et al., 2014; Pruim et al., 2015). For the remaining components, functional nodes’ time series were computed using dual regression technique (Filippini et al., 2009), and fed into temporal ICA.

For temporal ICA, we used Icasso algorithm (Himberg et al., 2004) to estimate the most appropriate decomposition yielding a set of reproducible IC clusters. For all possible dimensions or number of components, Icasso was ran with *both* resampling mode that uses random initial condition as well as bootstrapping of the data for 100 times, which pooled all temporal ICA estimates using FastICA (Hyvärinen, 1999) with tanh non-linearity and a symmetric decorrelation approach. We chose the decomposition yielding the maximum number of clusters of reproducible components that gives a stability index Iq larger than 0.5. Iq is computed as the difference between the average intracluster similarities and average intercluster similarities, which reflects the compactness and isolation of a cluster (Himberg et al., 2004). For each dataset, 28 and 30 reproducible clusters were found, respectively.

#### Identification of the Global Waves

For all temporal ICs, time series of each run, once concatenated to be subjected to a temporal ICA was deconcatenated so that following analyses can be performed for each run separately unless otherwise noted. Firstly, spatiotemporal patterns or paths of traveling waves were estimated for each IC by computing the relative time lag *t* that gives the best positive fit between each voxel’s time series and the time-shifted (±6.3 s or ±9 TR) IC (time series) using cross-correlation analysis. As in our previous study, 12 s limit of propagation delay was set to include whole-brain vascular time lag that can range up to nine seconds (Amemiya et al., 2016). Parabolic interpolation (Meijering, 2002) was further applied to locate the peak time lag *t’* using the extremum *t*, as well as the two nearest points given by the cross-correlation analysis.

The magnitude map of each IC was then computed as the Pearson’s correlation coefficients between each voxel’s time series and the IC time series that was shifted as much as *t’* using sinc interpolation to give the maximum correlation. Classification of ICs was based on the spatial distribution pattern of each component. We performed template matching using 21 RSN templates (Smith et al., 2012), as well as a whole-brain signal template that is the correlation map of the whole-brain mean signal, averaged over 100 runs (Supplementary Figure S1). The whole-brain signal was computed as the average signal within a gray matter mask was created by thresholding MNI template at 10% or larger probability of being gray matter. Pearson’s correlation coefficients were computed between each IC and each of the 22 templates within the mask. Any component that is more similar to the whole-brain signal template than any of the RSN templates (i.e., giving a greater correlation coefficient with a whole-brain signal template) was classified as global. Note that our study focuses particularly on examining the existence of coactivations restricted within each RSN, in addition to the globally propagating activities that were assumed to exist throughout the brain and treated as such in the analysis of previous studies. In this context, it would certainly make sense to identify any component whose distribution is restricted within any functionally distinct areas as being *local* as opposed to a functionally and spatially less specific *global* (or more precisely semi-global) pattern.

Using the time-shifted global ICs as regressors, linear regression analysis was performed for each voxel’s time series. The global component was then computed as the integral of all global ICs by summing up the shifted time series multiplied by the corresponding regression coefficients. The rest of the signal was classified as local contribution (Figure 1).

#### Estimation of the Likelihood That Each Mechanism Gives Rise to the RSN Synchronization

In order to evaluate the contribution of each mechanism to the emergence of RSN synchronization, we compared regions of interest (ROI)-wise correlation matrices given by each component using a set of 132 ROIs provided as part of the CONN toolbox (Whitfield-Gabrieli and Nieto-Castanon, 2012) that were originally defined from FSL Harvard-Oxford Atlas maximum likelihood cortical or subcortical atlas and cerebellar parcelation from AAL atlas.

For each subject’s each run, FC matrix was obtained by computing the Pearson’s correlation coefficient between each possible couple of ROI’s mean time series (i.e., global + local component). Similarly, three types of correlation matrices were computed by correlating the time series of (1) global component, (2) local component, (3) global component reconstructed to reflect only time lag of each global IC, respectively. We correlated each matrix against the FC matrix run-wisely, using the correlations above the diagonal of each matrix, transformed to Fisher’s Z and tested by using a two-tailed *t*-test over runs against the null hypothesis of no correlation. Next, we examined whether the time lag itself contributes to the RSN synchronization by computing the correlation between the FC matrix and the correlation matrix of the global component that was reconstructed without implementing the magnitude difference. In the presence of time lag, even when the global component is composed of a single IC, the spatial difference of its magnitude can contribute to the characterization of the correlation matrix, let alone the global component composed of multiple ICs. It is therefore important to eliminate the effect to determine if time lag is the source of synchronization. The magnitude of each IC was adjusted to reflect the contribution of each IC that is computed as the root mean square of the mixing matrix of the temporal ICA.

In order to further confirm the relationship between the signal synchronization and the time lag of the global component, for each global component, Pearson’s correlation coefficient was computed between the FC matrix and the matrix of relative time lag that is the difference of the time lags between given ROIs.

Contribution of the local component was also assessed by computing the correlation between the FC matrix and the correlation matrix of the local component in the same way. Correlation between the correlation matrices were computed using the upper triangle of each correlation matrix. The threshold of the statistical significance was set at *p* = 0.05, and the Bonferroni correction was used to control for the multiple comparisons.

#### Origin of the Time Lag

To estimate the origin of the time lag that characterizes the global component, all magnitude (correlation coefficient) and time lag maps of the global ICs that were averaged across subjects were compared with those of the whole-brain signal, respectively. Partial correlation analysis was also performed to control the effect of vascular time lag that was measured using dynamic susceptibility contrast enhanced perfusion imaging (Amemiya et al., 2016). We also compared the time lag structure of the local component and that of the whole-brain signal by computing the Pearson’s correlation between the time lag maps with each local IC. A correction for the spatial degrees of freedom was given via Gaussian random field theory and empirical smoothness estimation, which estimated the number of independent resels or resolution elements to be 103.

## Results

### Identification of the Global Waves

**FIGURE 2:**
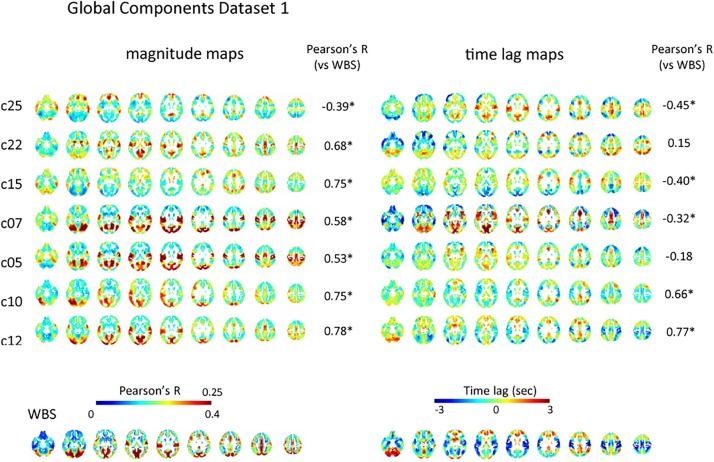
Global component. The spatial distribution of the magnitude of the temporal ICA components for test dataset demonstrates a set of global ICs. The magnitude of the seven global ICs is shown as Pearson’s correlation coefficient with the corresponding time lag structures showing its path. All magnitude maps were significantly correlated with the map obtained using the whole-brain signal (WBS), that is characterized by symmetrical high magnitude areas distributed predominantly in the occipital lobes, as well as in the path of propagation that is characterized by early signal in the primary sensorimotor and visual cortex compared with association areas, frontotemporal basal regions or the cerebellum (bottom row). Five of the seven time lag maps were significantly correlated with that of the whole-brain signal. C25, c07, and c05 show early regions in the rostral and lateral part of the frontal lobes and delayed regions in the medial part of the frontal lobes, insular and inferior frontal gyrus and occipital lobes, while the pattern is almost opposite for c10. C12’s path is characterized by early regions in the sensorimotor, auditory, and visual cortex, as well as delayed regions in the association cortex and posterior cingulate cortex, while c07 shows the opposite pattern. C15 resembles c05, c07, c25 pattern, but the delay in dorsal attention network is more conspicuous. Pearson’s correlation coefficients between time lag and magnitude maps of each global IC and those of the whole-brain signal are also shown.

Of the 28 and 30 reproducible ICs for dataset 1 and 2, 7, and 10 were classified as global IC based on the pattern of spatial distribution for each dataset, respectively (Figure 2 and Supplementary Figure S2). The magnitude of the global IC is shown as Pearson’s correlation coefficient between the time-shifted global IC and the time series of each voxel, with the corresponding time lag structures showing the paths of each global component (Figure 2 and Supplementary Figure S2). All magnitude and time lag maps shown were obtained by averaging the resulting maps across all subjects’ all runs. Time lag maps of the global components showed structural paths of each signal, which is consistent with previous studies suggesting the existence of multiple global waves of activity in the resting state; c25, c07 and c05 show early regions in the rostral and lateral part of the frontal lobes and delayed regions in the medial part of the frontal lobes, insular and inferior frontal gyrus and occipital lobes, while the pattern is almost opposite for c10. C12’s path is characterized by early regions in the sensorimotor, auditory, and visual cortex, as well as delayed regions in the association cortex and posterior cingulate cortex, while c07 shows the opposite pattern. C15 resembles c05, c07, c25 pattern, but the delay in the dorsal attention network is more conspicuous (Figure 2).

However, there were significant correlations among the paths of global signals (Supplementary Figures S6A,B). Some global ICs also showed apparent similarity to the whole-brain signal not only in its spatial distribution characterized by symmetrical involvement of the dorsal cerebral cortex with predominantly high magnitude in the occipital lobes, but also in its propagation pattern: time lag: |*r*| = 0.42 ± 0.21 (re-test, 0.37 ± 0.23); magnitude: *r* = 0.58 ± 0.15 (re-test, 0.55 ± 0.12) (Figure 2 and Supplementary Figure S2). Partial correlation analysis controlled for the perfusion time lag also showed sometimes reduced but still significant correlation between the time lag maps of the global ICs and the whole-brain signal: |*r*| = 0.40 ± 0.23 (re-test, 0.39 ± 0.22). These results suggest that even if multiple global waves of activity coexist in the resting state, the paths of the traveling waves can be substantially overlapped, as can the spatial distribution, which is not simply explained as the result of common background vascular perfusion.

Time lag maps of the global ICs detected from the test dataset were well replicated by the analysis of the re-test dataset. Supplementary Figure S6C demonstrates that all global ICs of the test data were significantly positively correlated with at least one global IC of the re-test data.

Consistent with previous studies (Mitra et al., 2014; Amemiya et al., 2016), magnitude and latency of the whole-brain signal were not significantly correlated: *r* = −0.10, *p* = 0.32 (re-test, *r* = −0.051, *p* = 0.62). The majority of the global components showed significant correlation between magnitude and time lag (*p* < 0.05): c07, *r* = 0.41; c10, *r* = −0.30; c12, *r* = −0.48; c12, *r* = −0.48; c25, *r* = −0.27 (re-test, c05, *r* = −0.29; c07, −0.44; c12, *r* = −0.31; c18, *r* = −0.36; c22, *r* = −0.24; c24, *r* = −0.25; c26, *r* = −0.42), which might be caused by the attenuation of the waves of activity during the process of transmission.

Some of the global ICs showed anteroposterior propagation that might correspond to the pattern detected using electroencephalogram in sleeping humans: c22 of the test dataset as well as c22 and c26 of the re-test dataset show early regions in the rostral compared with caudal part of the cerebral cortex.

### Contribution of the Global Waves to the RSN Synchronization

**FIGURE 3:**
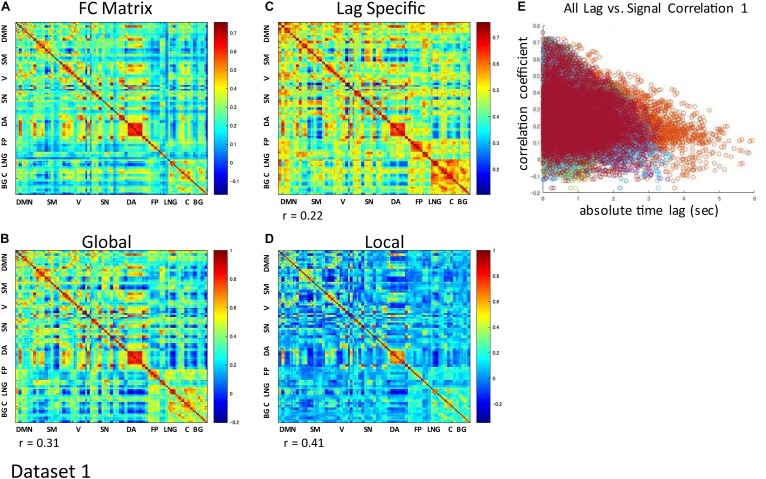
Contribution of each component to RSN synchronization. Whole signal Functional Connectivity (FC) matrix had significant positive correlation with the global component correlation matrix (**A** vs. **B**), suggesting the contribution of the global component to the RSN synchronization. Significant correlation is also shown even when the global component was reconstructed without considering the spatial difference of its magnitude (**A** vs. **C**). Correlation matrix of the local component is also fairly similar to the FC matrix, suggesting an equivalent contribution of locally limited activity to RSN synchronization (**A** vs. **D**). For each global component, there was significant negative correlation between the strength of synchronization (FC) and the relative time lag between each ROI, which confirmed the contribution of the time lag of the global component to RSN synchronization **(E)**.

**FIGURE 4:**
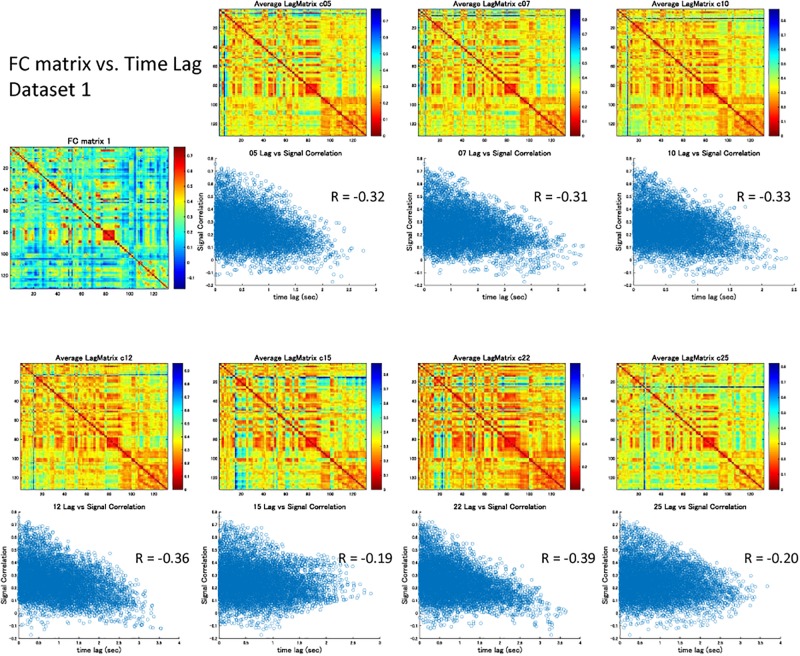
Signal correlation matrix vs. Time lag matrix. For each global component, there was significant negative correlation between the strength of synchronization (FC) and the relative time lag between each ROI, which confirmed the contribution of the time lag of the global component to RSN synchronization.

The correlation matrix of the global component reconstructed with the detected global ICs showed significant correlation with the FC matrix: *r* = 0.31 ± 0.13 (re-test, 0.32 ± 0.11), *p* < 0.001 (Figures 3A,B and Supplementary Figures S3A,B). Significant correlation was found even when the global component was reconstructed without considering the spatial difference of its magnitude: *r* = 0.22 ± 0.13 (re-test, 0.27 ± 0.11), *p* < 0.001 (Figure 3C and Supplementary Figure S3C). Furthermore, significant negative correlation between the strength of synchronization (FC) and the relative time lag was also shown for all global waves: *r* = −0.19 to −0.39, *p* < 0.001 (re-test, *r* = −0.14 to −0.40, *p* < 0.001) (Figures 3E, 4 and Supplementary Figures S3E, S4), which suggests that the time lag of the global component can contribute to the RSN synchronization.

### Characteristics of the Local Component

**FIGURE 5:**
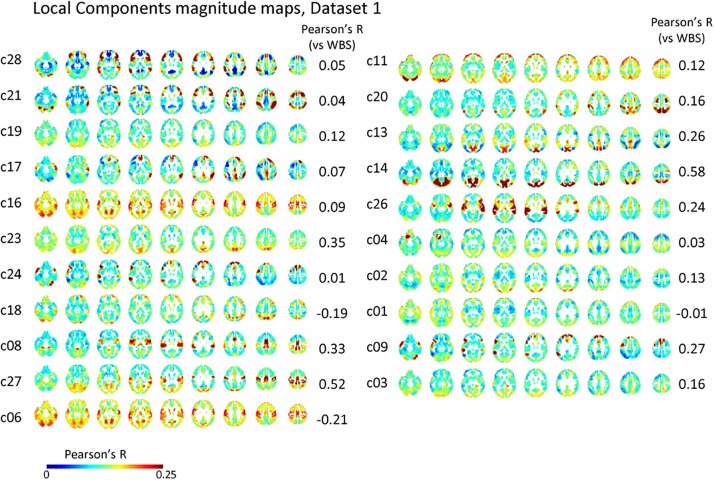
Local component. The spatial distribution of the magnitude of the temporal ICA components for test dataset demonstrates a set of local ICs. Each IC shows significant synchronization within functionally relevant structures that would correspond to spatial maps for the temporally independent functional modes (Smith et al., 2012).

The spatial distribution of the magnitude of the 28 and 30 reproducible local ICs is shown in Figure 5 and Supplementary Figure S5, respectively. Each magnitude map of the local ICs showed significant synchronization within functionally relevant structures, which would correspond to spatial maps for the temporally independent functional modes (Smith et al., 2012). In other words, as previously well-explored in Smith et al. (2012), the local ICs could be considered as functional “modes” that in some cases could subdivide and/or reorganize the currently standard spatial RSNs.; e.g., c01-03 contain visual cortex (predominantly extrastriate areas) and ventral sensorimotor cortex; c04 contains right orbitofrontal cortex in addition to sensorimotor and visual cortex; c06 contains extrastriate cortex and basal ganglia; c08 contains sensorimotor cortex, c09, c17, c18 involve frontotemporal network nodes; c11 mainly involves association cortex; c13 and contains primary visual cortex and default mode network nodes in the angular gyrus and posterior cingulate cortex; c14 contains visual cortex and dorsolateral prefrontal cortex; c16 involves relatively widespread areas mainly involving the sensorimotor and primary visual cortex in addition to basal ganglia; c19 contains; c20 and c21 contain dorsal attention network and frontal lobes; c23 involves primary visual cortex and frontal lobes; c24 and c28 contains salience network and frontoparietal network nodes; c26 contains auditory and sensorimotor networks; c27 involves sensorimotor and visual cortex. Figure 3D and Supplementary Figure S3D show correlation matrices of the local component that are fairly similar to the FC matrix (*r* = 0.41, *p* < 0.05; re-test, *r* = 0.42, *p* < 0.05), suggesting an equivalent or even larger contribution of the local activity to the RSN synchronization that characterizes the FC matrix. The magnitude of the activity of the local component relative to the whole signal was 0.70 for both test and re-test dataset.

## Discussion

By applying temporal ICA to the rs-fMRI data, we have identified several global or semi-global waves of slow oscillation that are temporally independent yet spatially overlapping with each other. Although the correlation matrix of the global component showed substantial correlation with the FC matrix, an equivalent or even greater contribution of the local component was also shown. The results indicate that while global waves of activity, although being less spatially homogenous than what was assumed to be, could contribute to the emergence of the RSN, which is partly consistent with previous studies suggesting that within-network synchronization can arise from the time lag of the global waves (Mitra et al., 2015a; Matsui et al., 2016), this does not exclude the contribution of local activity that are more confined within functionally relevant structures.

### Multiple Waves of Activity

Although the number of the global IC was slightly varied depending on the dataset, there was substantial overlap among the global ICs detected within or across the temporal ICAs for test and re-test datasets. Moreover, the majority of the global waves detected across the temporal ICAs were significantly correlated with the path of the whole-brain (global mean) signal, which would be the most robust representation of the global signal. Partial correlation analysis that controlled for the effect of vascular time lag also confirmed that significant correlation was still found with the global waves. While these results might support the finding that the global neural activity has a predominant path of propagation (Matsui et al., 2016), they also suggest the existence of multiple overlapping paths of neural oscillations. Some paths of global ICs that share common features with those obtained in the previous studies: c25, c05, c07, c10, and c12 would correspond to the thread 2 reported in Mitra et al. (2015a) that shows a contrast between the rostral and lateral part of the frontal lobes vs. medial part of the frontal lobes, insular and inferior frontal gyrus and occipital lobes, while c15 to thread 8 (note that the polarity of the threads can be inverted). However, there were some other time lag structures representing the paths of propagating neural activity that were not obtained by merely decomposing the measured total time lag as multiple orthogonal components (i.e., threads in Mitra et al., 2015a) or as independent components (Amemiya et al., 2016). Specifically, some global ICs showed anteroposterior propagation that might correspond to the pattern detected using electroencephalogram in sleeping humans (Massimini et al., 2004) or with calcium imaging as well as BOLD imaging in anesthetized mice (Stroh et al., 2013; Matsui et al., 2016). While physiological basis or significance of such global activity remains to be known, all these data further support the view that spatiotemporal pattern of BOLD signal could reflect large-scale dynamics of underlying neuronal activity.

### Origin of the Time Lag and Synchronization

Given that cerebral vascular time lag is quite uniform across subjects (Amemiya et al., 2016), existence of multiple paths of traveling BOLD signal suggest the existence of multiple waves of neural signal (Mitra et al., 2015a; Amemiya et al., 2016). Although BOLD represents hemodynamic response to neural activity that is necessarily influenced by the characteristics of the underlying vasculature (Amemiya et al., 2012; Bandettini, 2014), the path of globally propagating activity has been shown to coincide with that of the neuronal calcium signal in mice (Matsui et al., 2016). Assuming that the same holds true for non-anesthetized awake human data, the propagating pattern of global oscillations that are characterized by structured and smooth gradation can be seen as corresponding to a gradual propagation via short-range corticocortical connections. In addition, small time lag observed between distant regions across RSNs may reflect the presence of a mechanism controlling the initiation of spreading activity, mediated via long-range connections in a rapid manner. Such activities might help integrate the spontaneous oscillation of the cortex across RSNs in the whole brain, which is analogous to the concept of synfire chains in synchronous mode (Abeles, 1982, 1991), in which groups of neurons are organized into chains, and the architecture enables precisely timed sequences of spikes to form a propagating wave of activity (Abeles, 1982, 1991; Diesmann et al., 1999).

Alternatively, it would also be possible to assume that the time lag of the global oscillations mainly reflects vascular dynamics for some ICs. Indeed, whole-brain signal and vascular perfusion are known to share similar spatiotemporal characteristics (Amemiya et al., 2014, 2016; Tong et al., 2017). It might be important to note, however, that the source of the time lag is not necessarily identical to the source of the signal, so even if the time lag were totally non-neural in origin, that does not mean that the origin of the global signal is non-neural. This is because perfusion time lag can also be reflected in the time lag of BOLD signal of neural origin (Roc et al., 2006; Amemiya et al., 2012). Therefore, for the ICs whose time lag maps are similar to that of perfusion, e.g., like whole-brain signal, a measurement that is independent of neurovascular coupling would be preferable and perhaps essential for a more precise prediction of the spatiotemporal profile of the underlying neural activity.

Nevertheless, even if the contribution of some global oscillations to the apparent network synchronization were an artifact (Tong et al., 2015), the results of the present study suggest that network-specific synchronization does exist besides such component, which is consistent with the growing evidence supporting the link between BOLD and electroencephalographic or magnetoencephalographic measures of resting state activity (Goldman et al., 2002; Yan et al., 2009; Brookes et al., 2011; Tagliazucchi et al., 2012). Moreover, the present study indicates that within-network synchronization is dependent on local neural activities that are temporally independent to each other, which necessitates the presence of a mechanism that would be conceived as network-specific pacemaker irrespective of the contribution of the global oscillations.

### Technical Issues

In the present study, two large HCP datasets acquired from the same 50 subjects were used to test the reproducibility of the analysis. In temporal ICA, each component’s independence is optimized for the axis of *time*. Therefore, the temporal dimensionality or timepoints of the data should be large enough. Although there is no good reason to assume that the number of timepoints should be as large as the number of voxels in original data, when the dimension was reduced during the process of group spatial ICA in advance to temporal ICA. Rather the problem lies in that it is generally difficult and practically impossible to know in advance how many timepoints are needed for an ICA, which is particularly dependent on the non-gaussianity of the data. This is why *post hoc* analysis is generally considered important to validate an ICA.

It is also theoretically apparent that higher spatiotemporal resolution is preferable for a better mapping of the spatiotemporal characteristic of the data. However, identification of the global component is not likely dependent on the spatiotemporal resolution of the BOLD fMRI. This is because ICs are classified according to the pattern of spatial distribution, which is not dependent on the temporal or spatial resolution itself let alone the speed of the traveling waves. Therefore, granting that the time lag maps would become more accurate if the sampling rate or spatial resolution of the data were increased, given that the maps obtained in the present study represent structured and highly similar patterns even across studies for the whole-brain signal (Amemiya et al., 2014; Mitra et al., 2014; Tong et al., 2017), such contribution would be negligible compared with other factors, at least for the range of the neural band of 0.01–0.1 Hz. The same holds true for the slice-time correction. HCP do not recommend us doing slice timing correction for the dataset, because while the effect of the slice timing correction is limited for the short TR (0.72s), slice timing correction interacts with movement correction in ways that have not ever been appropriately addressed in available tools. However, given the fact that it is impossible to align the subjects’ head in exactly the same position for every scan for all the subjects, and that the acquisition was performed by using simultaneous multislice imaging with a slice thickness of 2 mm, we consider that the small slice timing differences were expected to be canceled out during the course of spatial normalization and group averaging of the time lag maps, which was confirmed by high correlation among the whole-brain signal time lag maps.

For the preprocessing, we did not apply global signal regression (GSR). Although GSR is a useful process to remove physiological noise like motion artifact, it eliminates any global signal regardless of the origin and can distort the resulting connectivity or activation measures in a complex way (Saad et al., 2012; Gotts et al., 2013; Glasser et al., 2016, 2018; Tang et al., 2019). Therefore, GSR and related approaches still remain controversial. Given that the study aim is to understand the possible contribution of the global signal, we consider it important not to apply GSR for our analysis.

In the present study, detection of the global IC was based on the spatial distribution pattern of each component that was judged by template matching using RSNs as well as the magnitude map of the whole-brain signal, which enabled us to classify an IC into global or local component without setting a threshold for the spatial coverage of that component. Although intuitively, an IC showing a larger spatial coverage would be considered as a global component, such classification is practically very difficult, because there is no objective definition regarding the coverage of a global component, as was the case in the original definition of the RSNs.

Rather, it is important to note that the present study, like previous studies, focuses on identifying slow waves with fixed patterns of propagation. While such an approach is advantageous in exploring the most robust representation of the phenomenon, an analysis allowing more spatiotemporally complex and dynamically changing patterns of propagation will probably reveal a more precise picture of the inter-network activities that may contribute to the integration of the network-specific activities constituting the functional architecture of the brain.

## Data Availability Statement

The datasets for this study are openly available at https://www.humanconnectome.org/.

## Author Contributions

SA contributed to the conception and design of the study, performed the statistical analysis, and wrote the first draft of the manuscript. SA and HT contributed to the interpretation of the data. All authors contributed to the manuscript revision, and read and approved the submitted version.

## Conflict of Interest

The authors declare that the research was conducted in the absence of any commercial or financial relationships that could be construed as a potential conflict of interest.

## Footnotes

1 http://www.fil.ion.ucl.ac.uk/

2 https://afni.nimh.nih.gov/
