# Supplementary material for: Global vs. Network-Specific Regulations as the Source of Intrinsic Coactivations in Resting-State Networks
Source: Front Syst Neurosci. 2019 Oct 29;13:65. doi: 10.3389/fnsys.2019.00065 (PMC6829116; doi:10.3389/fnsys.2019.00065)
Supplement: Supplementary file 1 [file Data_Sheet_1.docx]

Supplementary Material

# Supplementary Figures

**Fig S1. RSN templates vs. whole-brain signal magnitude map**

**
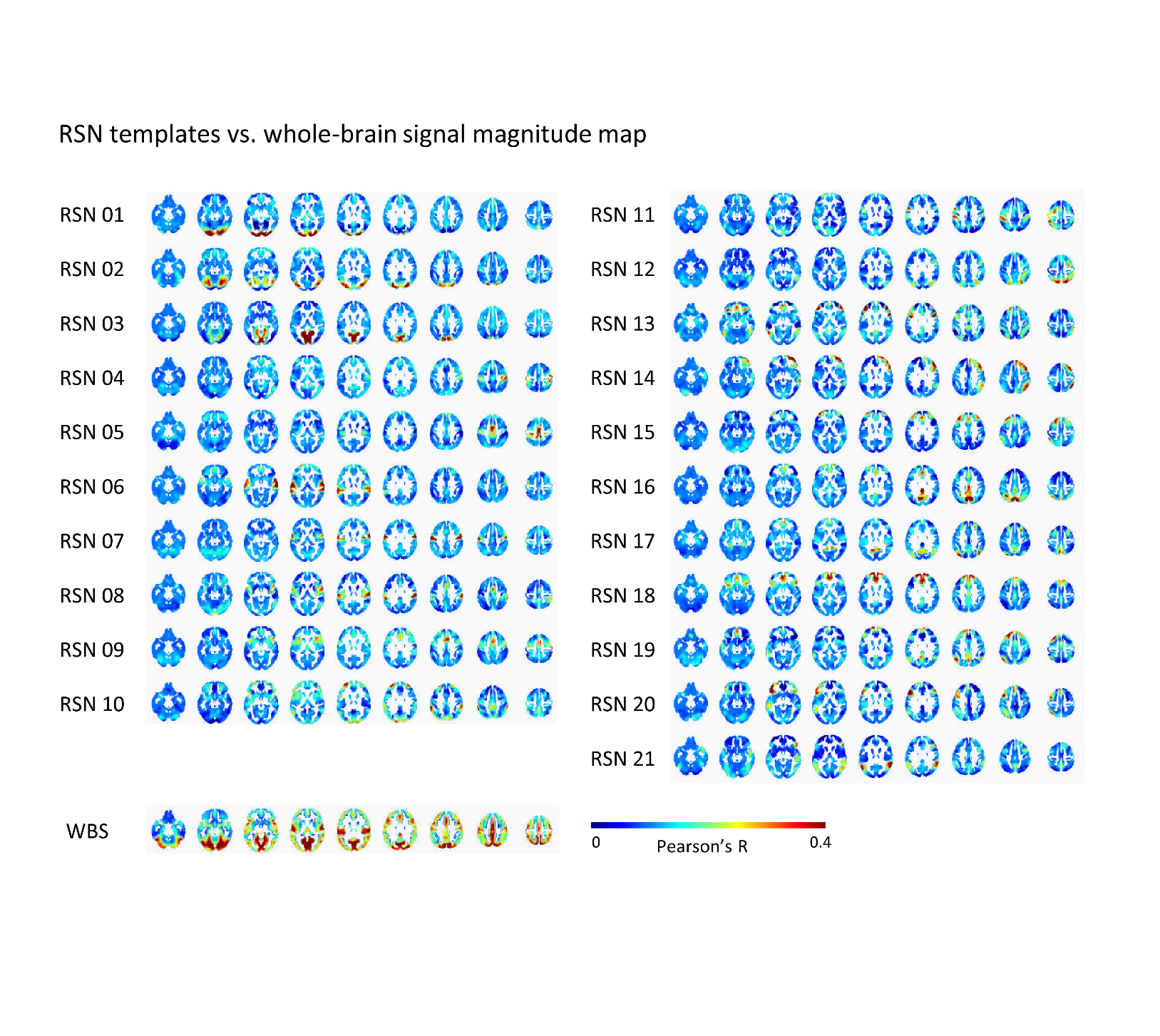
**

21 RSN templates and the magnitude map of the whole-brain signal (WBS) were used to judge if the spatial distribution of each IC is global or local. The unit of the RSN templates is arbitrary.

**
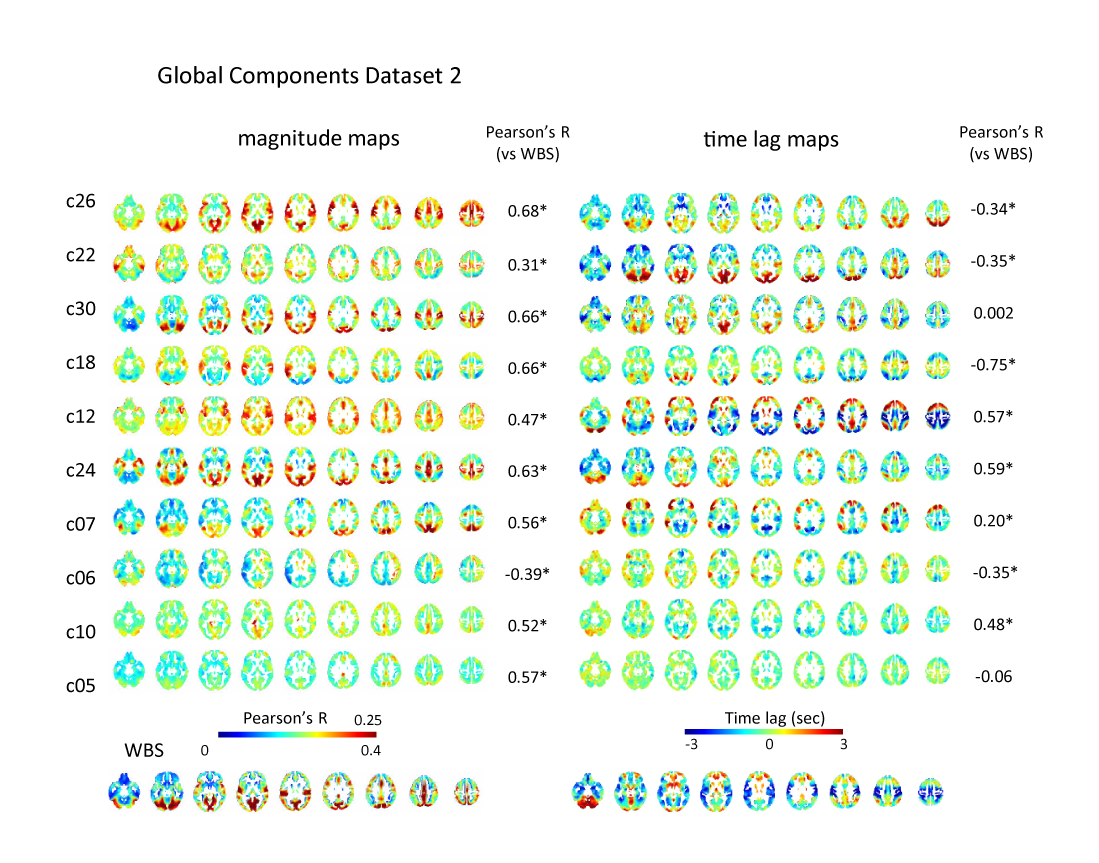
**

**Fig S2. Global component (re-test dataset)**

The spatial distribution of the magnitude of the temporal ICA components for re-test dataset demonstrates a set of global ICs. All magnitude maps were again, significantly correlated with the map obtained using the global mean signal (GMS). The majority of the time lag maps were significantly correlated with that of the whole brain signal (WBS). C12 and c24's paths are characterized by early regions in the sensorimotor, auditory, and visual cortex, as well as delayed regions in the association cortex and posterior cingulate cortex, while c06 and c18 shows the opposite pattern. C26 and c22 show early regions in the rostral compared with caudal part of the cerebral cortex. C07 and c10 resemble c12, c24 pattern, but the delay in frontoparietal network is more conspicuous. C30 shows early regions in the dorsal attention network and basal frontal regions. C05 is characterized by asymmetrical early and delayed regions in the frontoparietal network nodes.

##
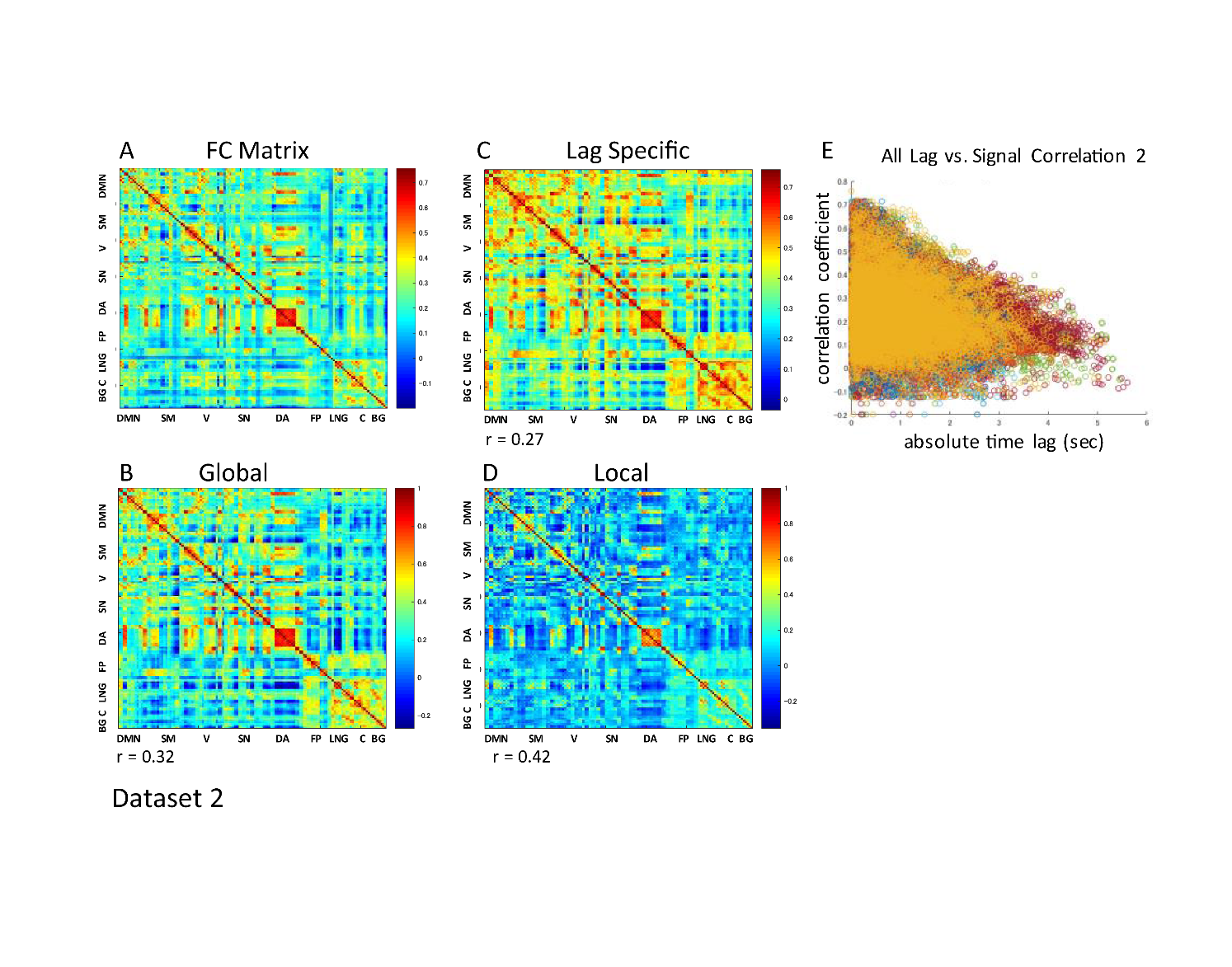


## Fig S3. Contribution of each component to RSN synchronization (re-test dataset)

The result of re-test dataset is similar to that of the test-dataset. Whole signal Functional Connectivity (FC) matrix had significant positive correlation with the global component correlation matrix (A vs B), suggesting the contribution of the global component to the RSN synchronization. Significant correlation is also shown even when the global component was reconstructed without considering the spatial difference of its magnitude (A vs C). Correlation matrix of the local component is also fairly similar to the FC matrix, suggesting an equivalent contribution of locally limited activity to RSN synchronization (A vs D). For each global component, there was significant negative correlation between the strength of synchronization (FC) and the relative time lag between each ROI, which confirmed the contribution of the time lag of the global component to RSN synchronization (E).

**
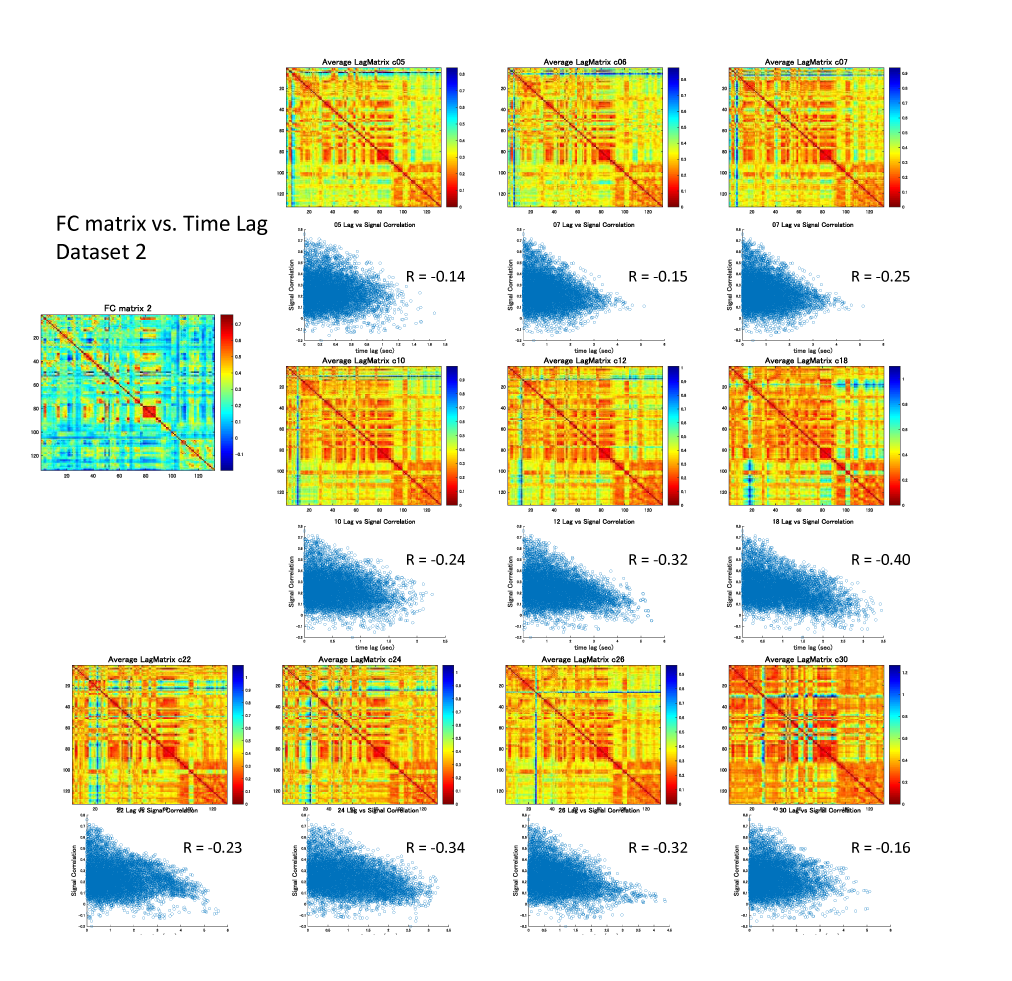
**

## Fig S4. Signal correlation matric vs Time lag matrix (re-test dataset)

The result of re-test dataset is similar to that of the test-dataset. For each global component, there was significant negative correlation between the strength of synchronization (FC) and the relative time lag between each ROI, which confirmed the contribution of the time lag of the global component to RSN synchronization (E).

**
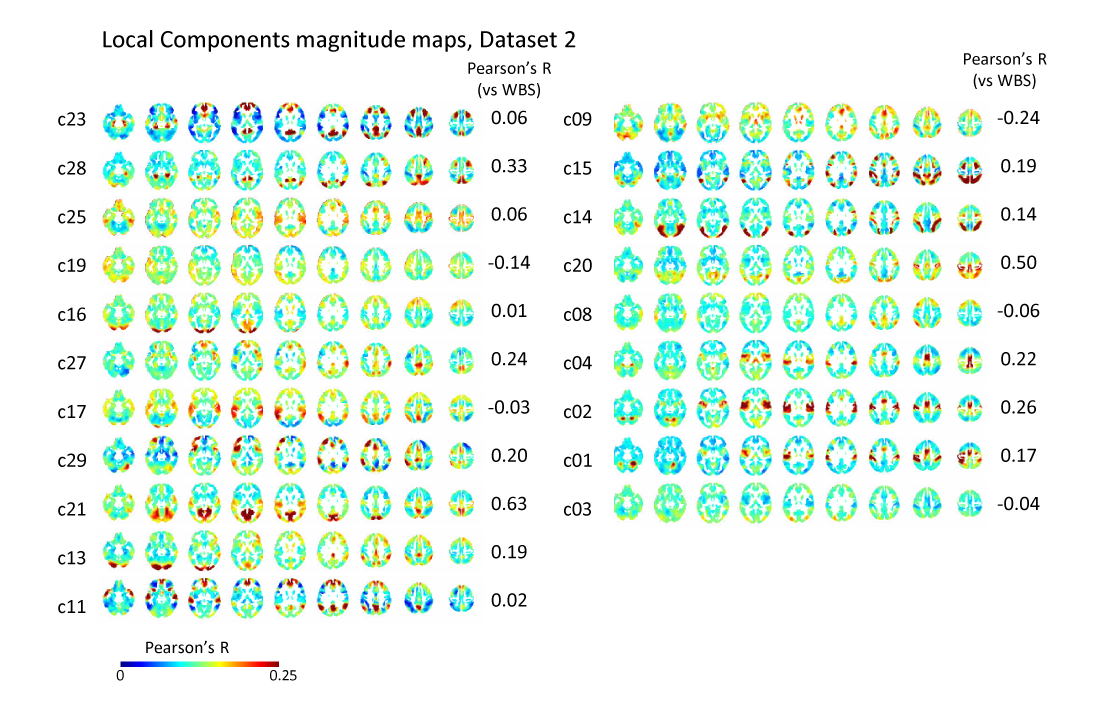
**

## Fig S5. Local component (re-test dataset)

The result of re-test dataset is similar to that of the test-dataset. The spatial distribution of the magnitude of the temporal ICA components for re-test dataset demonstrates a set of local ICs. Each IC shows significant synchronization within functionally relevant structures that would correspond to spatial maps for the temporally independent functional modes (Smith et al., 2012). Unlike global ICs, local ICs showed less-structured time lag distribution that had mostly low correlation with that of global mean signal.

**
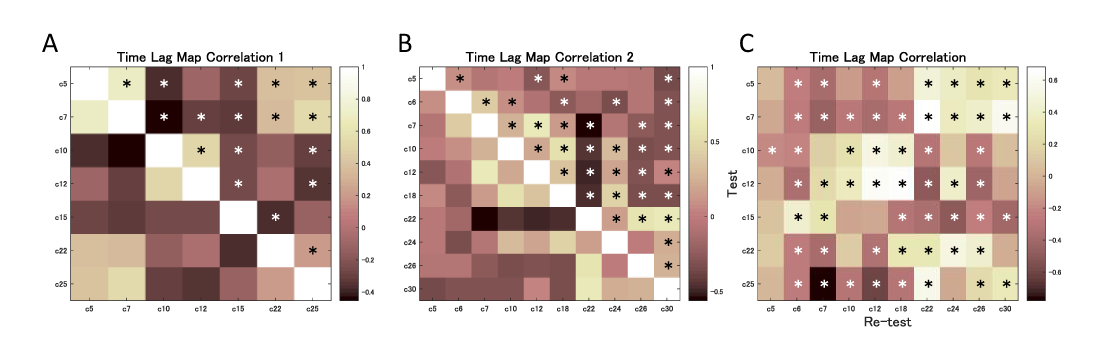
**

## Fig S6. Overlap among the time lag maps of the global ICs

There was substantial overlap among the time lag maps of the global ICs for test (A) and re-test (B) dataset respectively. C demonstrates that all global ICs of the test dataset show significant positive correlated with at least one global IC of the re-test dataset. Black asterisk indicates significant positive correlation, while white asterisk indicates significant negative correlation at a threshold of p < 0.05.
